# Experimental realization of a 3D random hopping model

**DOI:** 10.1038/s41467-021-27243-2

**Published:** 2021-11-30

**Authors:** Carsten Lippe, Tanita Klas, Jana Bender, Patrick Mischke, Thomas Niederprüm, Herwig Ott

**Affiliations:** Department of Physics and Research Center OPTIMAS, Technische Universität Kaiserslautern, 67663 Kaiserslautern, Germany

**Keywords:** Phase transitions and critical phenomena, Quantum simulation

## Abstract

Scientific advance is often driven by identifying conceptually simple models underlying complex phenomena. This process commonly ignores imperfections which, however, might give rise to non-trivial collective behavior. For example, already a small amount of disorder can dramatically change the transport properties of a system compared to the underlying simple model. While systems with disordered potentials were already studied in detail, experimental investigations on systems with disordered hopping are still in its infancy. To this end, we experimentally study a dipole–dipole-interacting three-dimensional Rydberg system and map it onto a simple XY model with random couplings by spectroscopic evidence. We discuss the localization–delocalization crossover emerging in the model and present experimental signatures of it. Our results demonstrate that Rydberg systems are a useful platform to study random hopping models with the ability to access the microscopic degrees of freedom. This will allow to study transport processes and localization phenomena in random hopping models with a high level of control.

## Abstract (web summary)

Three-dimensional spin models with random hopping disorder are relevant to a large variety of physical systems. Here, the authors present an experimental realization of such a model in a Rydberg system with dipole-dipole coupling and show signatures of a localization-delocalization transition.

## Introduction

Randomness and disorder have a strong impact on transport processes in quantum systems and phenomena such as Anderson localization^1–3^, many-body localization^4^ or glassy dynamics^5^ occur. Their characteristics depend on the strength and type of disorder. An important class are hopping models, where particles or excitations move through a system that has randomized couplings. This includes, e.g., spin glasses^5^, coupled optical waveguides^6^, or NV center arrays^7^. They are also important to understand excitation transport in molecular and biological systems, such as light-harvesting complexes^8^. In many of those systems, the microscopic coupling mechanism is provided by the dipole–dipole interaction. Rydberg systems with their strong and controllable dipolar interactions^9^ are thus an ideal platform to study energy transport in such random hopping models^10^.

A model particularly relevant to particle and energy transport is the XY model^11^ that describes coupled two-dimensional spin-$\frac{1}{2}$ particles by the Hamiltonian

1 $${\hat{H}}_{XY}=\mathop{\sum }\limits_{i < j}^{n}\frac{{J}_{ij}}{2}\left({\hat{\sigma }}_{i}^{x}{\hat{\sigma }}_{j}^{x}+{\hat{\sigma }}_{i}^{y}{\hat{\sigma }}_{j}^{y}\right)+\mathop{\sum }\limits_{i=1}^{n}{\varepsilon }_{i}{\hat{\sigma }}_{i}^{z},$$

where ${\hat{\sigma }}_{i}^{x/y/z}$ denote the Pauli matrices and *J*_*ij*_ describes the coupling between spins *i* and *j*. Rewriting the term $({\hat{\sigma }}_{i}^{x}{\hat{\sigma }}_{j}^{x}+{\hat{\sigma }}_{i}^{y}{\hat{\sigma }}_{j}^{y})/2={\hat{\sigma }}_{i}^{+}{\hat{\sigma }}_{j}^{-}+{\hat{\sigma }}_{i}^{-}{\hat{\sigma }}_{j}^{+}$ by the ladder operators emphasizes the hopping character of this model. The second term describes the on-site energy *ε*_*i*_.

For a realistic description of transport processes in solids, the inclusion of defects and disorders was found to be crucial. Adding disorder to the on-site energy *ε*_*i*_^1,12^ and restricting the coupling to nearest neighbors only results in the Anderson model, which laid the foundations to understand the metal–insulator transition. Several extensions to the Anderson model have been considered since then. Most prominently, the additional inclusion of on-site interactions led to the rapidly growing field of many-body localization^4,13^. Also, models with random on-site energy and long-range interaction were found to show many-body localized states^14,15^.

Another class of disordered systems described by Eq. (1) are hopping models with randomized couplings *J*_*ij*_. Such models have received broad interest due to a large variety of emerging effects such as many-body relaxation dynamics^16^, glassy dynamics^17^, localization phenomena^18–21^, or superfluid stiffness^22^. In many systems, random hopping is a consequence of position disorder. Ultracold Rydberg gases with their disordered particle distribution and characteristic power-law interaction are therefore an ideal testbed to study randomized hopping models. Here, we experimentally study a three-dimensional many-body Rydberg system with random dipole–dipole couplings. We measure the spectrum of the many-body system and compare it to an effective spin model. We discuss the appearance of a localization–delocalization crossover within our effective spin model and present experimental indications of such a transition. Our results pave the way to study transport processes and localization phenomena in random hopping models in detail.

The resonant dipole–dipole interaction between two atoms in Rydberg states of opposite parity, say an S- and a P-state, is the key ingredient to realizing an effective XY exchange interaction. The effective spin is encoded in the two Rydberg states $\left|{{{{{{{\rm{S}}}}}}}}\right\rangle \equiv \left|\downarrow \right\rangle$ and $\left|{{{{{{{\rm{P}}}}}}}}\right\rangle \equiv \left|\uparrow \right\rangle$. In the simple two-level system, the anisotropic dipole–dipole interaction ${\hat{V}}_{ij}^{dd}=({\hat{{{{{{{{\bf{d}}}}}}}}}}_{i}\cdot {\hat{{{{{{{{\bf{d}}}}}}}}}}_{j}-3({\hat{{{{{{{{\bf{d}}}}}}}}}}_{i}\cdot {{{{{{{{\bf{e}}}}}}}}}_{R})({\hat{{{{{{{{\bf{d}}}}}}}}}}_{j}\cdot {{{{{{{{\bf{e}}}}}}}}}_{R}))/{R}_{ij}^{3}$ leads to an XY spin-exchange term^23^ with the couplings ${J}_{ij}={C}_{3}(1-3{\cos }^{2}{\theta }_{ij})/{R}_{ij}^{3}$, where *R*_*ij*_ is the distance between the two atoms and *θ*_*ij*_ is the angle between the quantization and the interparticle axis. The system is governed by the Hamiltonian

2 $$\hat{H}=\mathop{\sum }\limits_{i < j}^{N}\frac{{C}_{3}\left(\theta \right)}{{R}_{ij}^{3}}\left({\hat{\sigma }}_{i}^{+}{\hat{\sigma }}_{j}^{-}+{\hat{\sigma }}_{i}^{-}{\hat{\sigma }}_{j}^{+}\right)+\mathop{\sum}\limits_{\nu =\downarrow ,\uparrow }\mathop{\sum }\limits_{i < j}^{N}\frac{{C}_{6}^{\nu }(\theta )}{{R}_{ij}^{6}}{\hat{n}}_{i}^{\nu }{\hat{n}}_{j}^{\nu },$$

where ${\hat{n}}_{i}^{\downarrow /\uparrow }=(1\pm {\hat{\sigma }}_{i}^{z})/2$ counts the number of $\left|\downarrow \right\rangle$/$\left|\uparrow \right\rangle$-excitations on site *i*. To a much lesser extent, the Rydberg system also realizes an Ising-type term through the van der Waals interaction between two identical spins $\left|\downarrow \downarrow \right\rangle$ or $\left|\uparrow \uparrow \right\rangle$, i.e., ${U}_{ij}={C}_{6}(\theta )/{R}_{ij}^{6}$^[ 24^. This correction is described by the second term in Eq. (2).

**Fig. 1:**
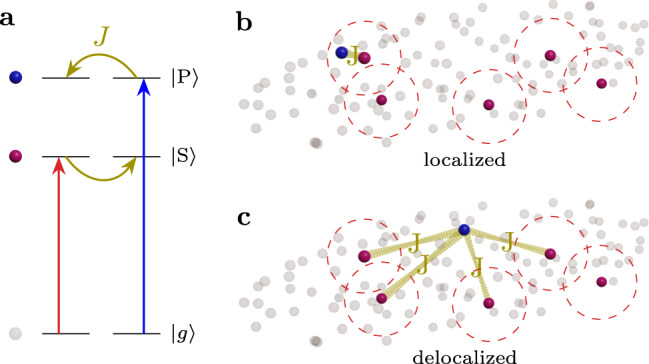
Sketch of the experiment. **a** Dipole–dipole interaction between two atoms. One ground state atom $\left|g\right\rangle$ (gray) is excited to a Rydberg $\left|{{{{{{{\rm{S}}}}}}}}\right\rangle$-state (red) with a two-photon transition (red arrow). A second atom is excited to a Rydberg $\left|{{{{{{{\rm{P}}}}}}}}\right\rangle$-state (blue) with a single-photon transition (blue arrow). Hopping with strength *J* (yellow arrows) is induced by resonant dipole–dipole coupling between the two Rydberg states of opposite parity. **b**, **c** Spatial distribution of Rydberg excitations corresponding to localized and delocalized states. Seed atoms in the Rydberg $\left|{{{{{{{\rm{S}}}}}}}}\right\rangle$-state (red) are separated by the Rydberg blockade radius (red dashed circles). Probe excitations to the Rydberg $\left|{{{{{{{\rm{P}}}}}}}}\right\rangle$-state are shown in blue. Surrounding ground state atoms are shown in gray. Yellow connections illustrate the strongest hopping contributions. They can be restricted to two sites only, forming a localized dimer state (**b**), or to multiple similarly spaced sites, forming a delocalized state (**c**).

The experiments are performed in a three-dimensional frozen Rydberg gas without an underlying regular lattice structure (Fig. 1). Hence, the *R*^−3^ scaling allows that one atom is possibly coupled to many others. The couplings are not purely random, as the individual distances in a system with position disorder are still correlated due to the triangular inequality and Rydberg blockade. In the experiment, we realize the weak probing limit, where the *C*_6_ term of Eq. (2) effectively simplifies to a random field $\mathop{\sum }\nolimits_{i = 1}^{n}{\varepsilon }_{i}{\hat{\sigma }}_{i}^{z}$ and the Hamiltonian takes the form of the pure XY model Eq. (1) (see Supplementary Note 1). Note that with a proper choice of the involved Rydberg states, the relative strength of the two terms in Eq. (2) can be tuned, thus allowing to study the crossover from hopping disorder to on-site disorder (Supplementary Note 4).

## Results and discussion

### Experiment

**Fig. 2:**
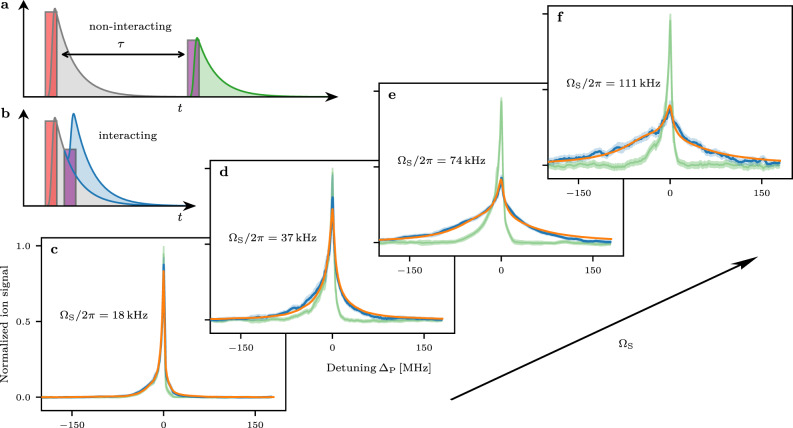
Spectroscopy of dipole–dipole coupled many-body Rydberg systems. **a**, **b** illustrate the pump–probe excitation scheme with delay *τ* in the non-interacting (**a**) and interacting case (**b**). The first pulse (red) creates seed excitations in the $|51{{{{{{{{\rm{S}}}}}}}}}_{1/2}\rangle$-state. The second pulse (purple) excites atoms to the $|51{{{{{{{{\rm{P}}}}}}}}}_{3/2}\rangle$-state. **c**–**f** Spectra for the excitation of the $|51{{{{{{{{\rm{P}}}}}}}}}_{3/2}\rangle$-state after the creation of different numbers of initial seeds in the $|51{{{{{{{{\rm{S}}}}}}}}}_{1/2}\rangle$-state with two-photon Rabi frequencies Ω_S_ (**c**) 2*π* × 18 kHz, (**d**) 2*π* × 37 kHz, (**e**) 2*π* × 74 kHz, (**f**) 2*π* × 111 kHz. The interacting (*τ* = 1 μs, blue) and non-interacting (*τ* = 300 μs, green) spectra in (**c**–**f**) are obtained by integrating the blue and green shaded area of the time-resolved signals in (**a**) and (**b**) for each detuning Δ_P_, respectively. The shaded areas in (**c**–**f**) denote the standard error of the mean. In the interacting case (**b**), the signal from the probe pulse (blue shaded area) is isolated by subtracting the pump signal (gray shaded area) from the non-interacting case (**a**) (see Supplementary Note 3 for details). The results of the numerical model are shown as orange lines. We extract average coherently coupled seed excitation numbers $\bar{n}$ of (**c**) 0.6, (**d**) 2.4, (**e**) 5.0, and (**f**) 5.7.

We study the Hamiltonian Eq. (2) spectroscopically. This gives access to the density of states of the many-body system and its scaling properties. This approach allows for a direct comparison with numerical simulations and helps us to identify signatures for the appearance of localized and delocalized states. The experimental realization requires a gas with a high number density in order to realize a high absolute number of small interatomic distances such that many closely spaced, strongly interacting pairs can be excited and detected. For this purpose, we prepare a Bose-Einstein condensate (BEC) of ^87^Rb (see Methods). The experiment is conducted in a pump–probe scheme (Fig. 2a, b). In the first step a variable number of atoms is brought into the $\left|\downarrow \right\rangle$-state (labeled as “seeds”) and a subsequent pulse excites on the order of one ground state atom into the $\left|\uparrow \right\rangle$-state. The excitation to the spin-down state $|\downarrow \rangle =|51{{{{{{{{\rm{S}}}}}}}}}_{1/2},{m}_{J}=1/2\rangle$ is realized by a 1 μs long resonant two-photon pulse. The average number $\bar{n}$ and the spatial distribution of seed excitations are controlled by the coupling strength Ω_S_ and the Rydberg blockade conditions^25,26^. After a variable delay time *τ*, we apply the 1 μs long probe pulse by weakly driving (Ω_P_ ≪ Ω_S_, detuning Δ_P_) a single-photon transition from $|5{{{{{{{{\rm{S}}}}}}}}}_{1/2}\rangle$ to the $|\uparrow \rangle =|51{{{{{{{{\rm{P}}}}}}}}}_{3/2},{m}_{J}=1/2\rangle$ Rydberg state. The spontaneous decay of Rydberg atoms into ions allows continuous and time-resolved probing of the Rydberg population. Without probe pulse, the seed excitations decay on a typical timescale of *τ*_eff_ ≲ 15 μs. The delay between the pump and the probe pulse is either chosen to be *τ* = 1 μs ≪ *τ*_eff_ to create an $\left|\uparrow \right\rangle$-excitation in the presence of the $\left|\downarrow \right\rangle$-seeds (interacting case) or it is chosen to be *τ* = 300 μs ≫ *τ*_eff_ to obtain a reference measurement of the temporally separated $\left|\downarrow \right\rangle$ and $\left|\uparrow \right\rangle$ excitations (non-interacting case). By changing the probe laser detuning, we observe the spectroscopic response of the $\left|\uparrow \right\rangle$-excitation in the presence of a variable number of seeds, thus probing the random XY model. Note that in our system, the typical dipole–dipole coupling strength between two Rydberg atoms is much larger than the estimated decoherence rate stemming from laser noise and intrinsic decay processes. The spectroscopy therefore predominantly probes the coherent many-body states (see Supplementary Note 2 for a quantitative estimate).

A series of interacting spectra for increasing pump power is shown in Fig. 2c–f together with a reference measurement. One can see that the spectroscopic line shape for the excitation of the $\left|\uparrow \right\rangle$-state significantly broadens. While for small Rabi coupling, we see only small deviations from the non-interacting case, the line shape becomes largely modified for strong pump powers. On the one hand, the *C*_3_ Rydberg blockade manifests through the suppression of the spectroscopic signal on resonance. On the other hand, the dipole–dipole-induced anti-blockade shows up as a strong enhancement of the signal far from resonance^27,28^. For small pump power, we statistically see many realizations without seed excitations. Here and in the reference spectra, the line shape shows an increased signal for negative detunings which can be attributed to the formation of ultralong-range Rydberg molecules^29^. In the presence of seeds, however, the molecule formation becomes strongly suppressed due to the reduced probability to find an atom that simultaneously has the proper distance to all seeds and a ground-state atom to form the molecule.

### Spin model simulation

To model our experimental spectra we perform Monte Carlo simulations of the random XY model Eq. (2). We restrict our treatment to the weak probing limit (Ω_P_ ≪ Ω_S_) and only consider the single-excitation subspace spanned by states $\left|i\right\rangle =\left|{{{{{{{{\rm{S}}}}}}}}}_{1},{{{{{{{{\rm{S}}}}}}}}}_{2},\ldots {{{{{{{{\rm{P}}}}}}}}}_{i}\ldots {{{{{{{{\rm{S}}}}}}}}}_{n}{{{{{{{{\rm{S}}}}}}}}}_{n+1}\right\rangle$ with the $\left|{{{{{{{\rm{P}}}}}}}}\right\rangle$-excitation sitting on position *i*. For a set of parameter combinations of the number of seed excitations *n* and the blockade radius *r*_*B*_ we numerically diagonalize the Hamiltonian Eq. (2) in this subspace for 10^5^ realizations and obtain the eigenstates $|{\xi }_{j}\rangle$ and eigenenergies *E*_*j*_. For a single realization *n*$\left|{{{{{{{\rm{S}}}}}}}}\right\rangle$-seeds obeying blockade conditions and an additional randomly positioned particle representing the $\left|{{{{{{{\rm{P}}}}}}}}\right\rangle$-excitation are drawn from the BEC density distribution. Also accounting for Poissonian fluctuations in the number of seeds and the appearance of Rydberg molecules in the absence of seeds, we obtain simulated spectra ${\bar{\chi }}_{\bar{n},{r}_{B}}({{{\Delta }}}_{{{{{{{{\rm{P}}}}}}}}})$ for an average seed number $\bar{n}$ and blockade radius *r*_B_ which are fitted to the measured spectra (see Methods).

The resulting fitted line shapes are shown in Fig. 2c–f. A remarkable quantitative agreement between calculated and measured spectra is achieved. Both effects, the suppression on resonance and the strong enhancement at large detunings, are recovered. The model also correctly predicts the small but noticeable asymmetry towards negative detunings. It can, therefore, not be attributed to the creation of molecules, as they are not included in the model (except to describe the influence of measurements without seeds in the weak pumping limit).

The asymmetry is also remarkable as the binary interaction of a single $\left|{{{{{{{\rm{S}}}}}}}}\right\rangle$- with a single $\left|{{{{{{{\rm{P}}}}}}}}\right\rangle$-excitation creates a strictly symmetric spectrum. However, beyond this binary regime, which has been studied previously^30–32^, the eigenspectrum itself gives rise to an asymmetry due to correlations in the hopping matrix elements^19^. This effect even prevails in the absence of the *C*_3_ angular dependence and the weak *C*_6_ interaction.

As expected, the fitted number of coherently coupled seeds $\bar{n}$ increases with the Rabi frequency of the pump pulse, up to $\bar{n}=5.7$. For the largest coupling, we, therefore, probe the simultaneous coherent interaction of one $\left|\uparrow \right\rangle$- with six $\left|\downarrow \right\rangle$-spins. Comparing $\bar{n}$ with an independent estimate based on the absolute number of detected ions agrees for small Rabi frequencies. For the highest prepared seed densities, however, we see deviations that might originate from fast redistribution processes like l-changing collisions into states not interacting with the probe excitation. Additionally, with increasing pump power the seed density saturates in the center of the cloud due to the Rydberg blockade, and an increasing fraction of seeds is created in the thermal wings where they are too far apart to become part of a coherent state. Compared with a model that does not include the *C*_6_ term in Eq. (2), we see that the van der Waals interaction only provides minor corrections to the spectral shape.

We also repeated the measurement using a different fine structure state $\left|{\uparrow }^{\prime}\right\rangle =|51{{{{{{{{\rm{P}}}}}}}}}_{1/2},{m}_{J}=1/2\rangle$ (see Supplementary Note 5). We find the same level of quantitative agreement, suggesting that the microscopic details of the atomic-level structure play a minor role and our system is adequately described by the effective two-level spin Hamiltonian Eq. (2). Throughout all measurements, we consistently obtain a blockade radius of *r*_*B*_ = 3.4 μm which fits well to the expected value.

### Localization–delocalization crossover

The question of localization in dipole–dipole interacting systems is subtle. For power-law hopping models in cubic lattices with random on-site energy, a critical dimension analysis reveals a hopping-induced breakdown of localization in 3D^15^. Recent studies show, however, that the addition of hopping disorder can restore localization^19,21^. In fact, the eigenstates are expected to show a crossover from a regime with predominantly delocalized states to pair-localized states^19^, depending on the energy of the state.

Having verified the validity of our effective spin model, we can address these questions for our system by looking at the structure of the eigenstates and their eigenenergies. We illustrate this procedure with two descriptive configurations that can occur. Since the position of the $\left|\uparrow \right\rangle$-atom is randomly chosen without any distance constraints, it can possibly be very close to one of the seeds (but not to more than one, due to the blockade between the seeds), as sketched in Fig. 1b. In this limit, two eigenstates $\left|{\xi }_{\pm }\right\rangle \approx 1/\sqrt{2}(\left|i\right\rangle \pm \left|j\right\rangle )$ exist at high positive and negative energy ± *J*_*i*,*j*_/2, where the $\left|\uparrow \right\rangle$-spin is localized on the closely separated pair {*i*, *j*}. In the other limit, all the distances between the spins are similar and the couplings between them are smaller. Moreover, as they are also comparable in size, interference of different paths becomes possible. As a consequence, a set of low-energy, highly delocalized states (Fig. 1c) emerges. Since the localized states exist predominantly at high energies and the delocalized states at low energies, the random hopping model is predicted to show a localization–delocalization crossover^18,19,21,33^. In these studies, indications for the existence of such a crossover have been found, based on level statistics^19^, or on the eigenstate properties^21^. However, an experimentally accessible criterion to identify the emergence of a localization–delocalization crossover was missing.

To develop a measure for the degree of localization of the eigenstates $|{\xi }_{j}\rangle$ we inspect their coherence

3 $$C(|{\xi }_{j}\rangle )=\mathop{\sum}\limits_{i}\mathop{\sum}\limits_{k\ne i}\left|{({c}_{i}^{j})}^{* }{c}_{k}^{j}\right|$$

for each eigenstate $|{\xi }_{j}\rangle =\sum {c}_{i}^{j}\left|i\right\rangle$. Intuitively, the coherence roughly gives the number of atoms coherently sharing the $\left|\uparrow \right\rangle$-excitation^34^. Its minimal value *C* = 1 correponds to a dimer state while the value *C* = *n* is reached for a maximally delocalized state with equal probability to find the $\left|\uparrow \right\rangle$-spin on any of the sites. In the following, we define an eigenstate with a coherence below a threshold *C* < *C*_thresh_ = 2 to be localized in Hilbert space. We checked that in our system, for system sizes *n* ≿ 3, localized states in Hilbert space also fulfill the notion of localization in real space. As we are also investigating smaller systems, which trivially fulfill *C* < 2, we extend the criterion for localization by a maximum allowed spatial extent given by the blockade radius (see Supplementary Note 6 and Supplementary Note 7).

**Fig. 3:**
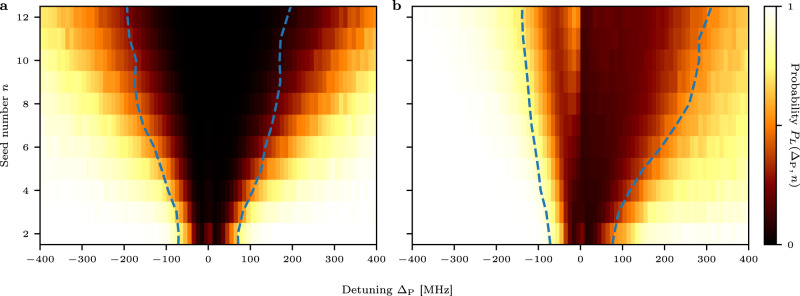
Localization–delocalization crossover. We plot the probability *P*_*L*_(Δ_P_, *n*) to find a localized state for (**a**) a pure random XY model and (**b**) with additional *C*_6_ interaction between the seeds as realized in the experiment. The regime of predominantly delocalized states spreads for an increasing number of seeds *n*, shifting the localization--delocalization crossover to larger energies. The estimated energies Δ_CO_ where the corresponding calculated spectra show a transition towards algebraic ∣Δ_P_∣^−2^ scaling are denoted by blue dashed lines. For a more detailed explanation of how Δ_CO_ is determined from the individual calculated spectra, see Methods for a mathematical derivation and Supplementary Fig. 6 for a graphical representation.

To identify a localization–delocalization crossover, we now calculate the conditional probability *P*_*L*_(Δ_P_, *n*) to find a localized state at a given excitation energy *E* for various numbers of seed excitations *n*. These probabilities *P*_*L*_(Δ_P_, *n*) for seed numbers up to *n* = 12 are depicted in Fig. 3a, b for the model Eq. (2) without and with the weak *C*_6_ interaction, respectively. As expected, the phase diagram clearly shows a crossover from delocalized to localized eigenstates when increasing the detuning. For a larger number of seeds *n*, the crossover shifts to higher energies. This can be understood by decreasing the average distances between the seeds. While the addition of the *C*_6_ interaction introduces only minor corrections to the spectral shape, the structure of the eigenstates changes substantially (Fig. 3b). Compared to the pure XY model, the transition is shifted towards blue detuning due to the repulsive nature of the *C*_6_ interaction. Additionally, the energy range, where the states are predominantly delocalized, shrinks. This originates from the weak probing limit, where the *C*_6_ interaction can be mapped to a random longitudinal field *ε*_*i*_. The spectral narrowing can therefore be interpreted as a manifestation of Anderson localization. This effect is even more prominent for the other fine structure state $\left|{\uparrow }^{\prime}\right\rangle =|51{{{{{{{{\rm{P}}}}}}}}}_{1/2},{m}_{J}=1/2\rangle$ considered in this work because the weaker dipole–dipole interaction of this state induces a larger relative strength of the random longitudinal field (see Supplementary Note 5).

**Fig. 4:**
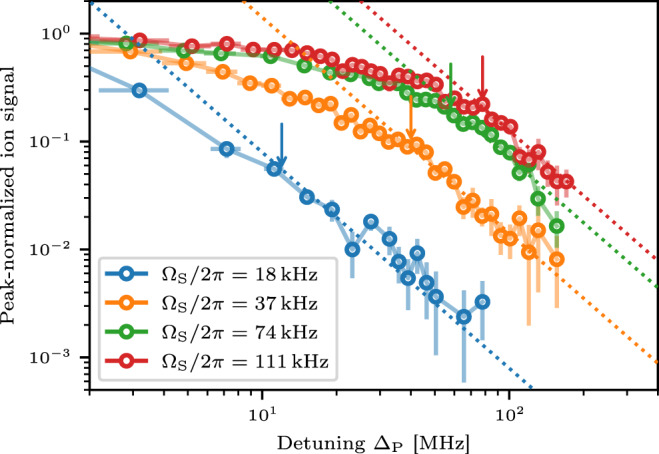
Scaling behavior of the interacting spectra. The measured spectral density (dots) approaches the expected ∣Δ_P_∣^−2^ scaling (dotted lines) for large detuning. This scaling indicates the occurrence of predominantly localized states. The arrows indicate the detuning where the transition toward ∣Δ_P_∣^−2^ scaling sets in. This corresponds to the dashed lines in Fig. 3. For increasing Rabi frequency Ω_S_ and thus increasing seed atom number the onset of the localization--delocalization crossover shifts to larger detunings. Horizontal error bars reflect energy bin sizes for the evaluation. Vertical error bars denote the standard error of the mean of the ion signal strength.

It is not possible to directly measure the coherence in our system. To gain an experimentally accessible observable, we investigate the spectral structure of the crossover. Previous studies have shown that localized pair states manifest in an algebraic decay in the tails of the spectral density *f*(∣Δ_P_∣ → *∞*) ∝ ∣Δ_P_∣^−2^^19,35^. Thus, for all the simulated eigenvalue spectra ${\chi }_{n,{r}_{B}}({{{\Delta }}}_{{{{{{{{\rm{P}}}}}}}}})$ we identify the energy Δ_CO_ where the scaling of the spectral density approaches ∣Δ_P_∣^−2^ scaling (Fig. 3, dashed lines). Indeed, Δ_CO_ qualitatively follows the equiprobability lines of *P*_*L*_(Δ_P_, *n*), rendering the change in scaling behavior an experimentally accessible indicator for the crossover towards localized states. An inspection of the experimental data (Fig. 4) shows the predicted ∣Δ_P_∣^−2^ scaling for large detunings. By comparison with the localization results from the model, we identify this asymptotic behavior with the crossover to the predominantly pair-localized regime. In accordance with our simulations and previous studies^19^, the energy where the crossover appears increases with the number of seeds. Compared to the simulations, the crossover happens already at smaller detunings. A possible reason could be, that the microscopic details of the excitation process are not fully captured by the classical rate model.

**Fig. 5:**
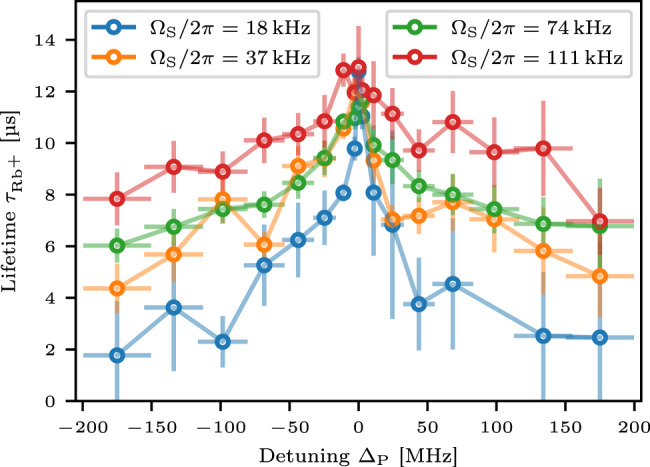
Lifetime ${\tau }_{{{{{{{{{\rm{Rb}}}}}}}}}^{+}}$ of the Rb^+^ ionization channel. With increasing detuning Δ_P_ the lifetime drops. Increasing the Rabi frequency Ω_S_ and thus increasing the seed atom number for a fixed detuning leads to a longer lifetime, indicating a larger fraction of delocalized states. The lifetimes are obtained numerically from the ion signal under the assumption of exponential decay of the Rb^+^ channel of the time-resolved spectra (see Supplementary Note 3). Horizontal error bars reflect energy bin sizes for the evaluation. Lifetime errors are given by error propagation of the standard error of the mean.

As a second indicator for localization, we study the lifetimes of the observed Rydberg states. Since high energetic dimer states are associated with small interparticle distances, they are subject to strong acceleration and rapid motion. The giant interaction cross-section of the moving Rydberg atoms with the surrounding dense bath of ground state particles leads to efficient ionization^36^. Due to the increasing fraction of pair-localized states, we thus expect the lifetime of the Rydberg excitations to decrease for increasing laser detuning. To this end, we analyze the lifetime ${\tau }_{{{{{{{{\rm{R{b}}}}}}}^{+}}}}$ of the Rb^+^ ion signal after the probe pulse. Figure 5 shows that ${\tau }_{{{{{{{{\rm{R{b}}}}}}}^{+}}}}$ decreases with increasing laser detuning, signaling the rising contribution of localized states. For a higher number of seeds the delocalized states dominate over an increasingly large energy range. Thus, the extracted lifetimes drop slower with energy as the pumping strength is increased. Both experimental findings, the asymptotic ∣Δ_P_∣^−2^-scaling of the spectra as well as the reduced lifetime of the Rydberg excitations, suggest that the system exhibits a localization–delocalization crossover.

Interacting Rydberg gases are an almost perfect model system to study the interplay between long-range interactions, random hopping and localization. Future extensions of our studies are straightforward: the relative strength of the hopping term and the onsite energy can be tuned by a proper choice of the involved Rydberg states. The recent advent of tweezer arrays provides ideal conditions to look in detail at the emerging spatial structure of the localized states. Their perfect control over each and every single atom even in 3D^37^ allows the reproducible creation of tailored disordered patterns. The inclusion of local excitation and readout processes would open up additional ways of probing the microscopic physics of transport dynamics in the context of open quantum systems. Eventually, strong correlations are realized in the strong probing limit, where a comparable number of both spin states is present in the sample.

## Methods

### Experimental procedure

Starting from a 3D magneto-optical trap, we prepare a Bose–Einstein condensate of ≈90 × 10^3^ ^87^Rb atoms with a peak density of 3 × 10^14^ cm^−3^, spin polarized in the $|5{{{{{{{{\rm{S}}}}}}}}}_{1/2},F=2,{m}_{F}=2\rangle$ ground state, by performing forced evaporative cooling in a crossed YAG dipole trap with final trapping frequencies *ω*_*r*_ ≈ 2*π* × 160 Hz and *ω*_*a*_ ≈ 2*π* × 90 Hz in radial and axial direction, respectively. The $|51{{{{{{{{\rm{S}}}}}}}}}_{1/2},{m}_{J}=1/2\rangle$ seed excitations are created with a two photon transition using a combination of continuous-wave lasers at 420 and 1015 nm both driving a *π*-transition. Due to a large blue detuning Δ_int_ = 160 MHz to the intermediate $|6{{{{{{{{\rm{P}}}}}}}}}_{3/2}\rangle$ state, it can be adiabatically eliminated, allowing to describe the excitation with an effective Rabi frequency Ω_S_. The power of the infrared coupling laser is kept constant at 450 mW with a 1/*e*^2^ diameter of 150 μm, the power of the weak blue beam (1/*e*^2^ diameter of 1.7 mm) is varied to set effective Rabi frequencies Ω_S_ between 2*π* × 18 and 2*π* × 111 kHz. The coupling of the ground state with the $|51{{{{{{{{\rm{P}}}}}}}}}_{3/2},{m}_{J}=1/2\rangle$ state is generated by a frequency-doubled continuous-wave dye laser at 297 nm with a 1/*e*^2^ diameter of 100 μm driving a *π*-transition. The Rabi frequency is fixed to Ω_P_ ≈ 2*π* × 4.5 kHz. The quantization axis is set along the vertical axis, i.e., along a radial direction of the optical dipole trap. Both pump $\left|{{{{{{{\rm{S}}}}}}}}\right\rangle$- and probe $\left|{{{{{{{\rm{P}}}}}}}}\right\rangle$-excitation pulses have a duration of 1 μs. The probe pulse either occurs at a delay *τ* = 1 μs or *τ* = 300 μs after the pump pulse, corresponding to the interacting and non-interacting case, respectively. After *τ* = 1 μs, only 6% of the seeds are decayed and only a fraction of those into ions. The presence of ions during the probe pulse can thus be neglected. Using a small electric field (*E* ≈ 50 mV cm^−1^) we continuously guide the few ions created through intrinsic ionization processes of the Rydberg atoms^36^ to a discrete dynode detector. This allows us to record a time-resolved ion signal proportional to the Rydberg population^38^. Every 600 μs we repeat the pulse sequence for 1000 repetitions in total before a new sample is prepared. We evaluate only the first repetitions until the density of ground-state atoms drops to ≈2/3 of its initial value.

### Simulations

For the numerical spin model simulation, we initialize an ensemble with random particle positions obeying a Thomas-Fermi distribution (*N* = 90 × 10^3^ and Thomas–Fermi radii *r*_TF_ = (4.6, 8.2, 4.6) μm). While due to the complex ionization channels, the exact number of created $\left|\uparrow \right\rangle$-excitations is hard to determine precisely, we estimate it to be on the order of one. Thus, we restrict our treatment to the weak probing regime (Ω_P_ ≪ Ω_S_) and only consider the single-excitation subspace spanned by the states $\left|i\right\rangle =\left|{{{{{{{{\rm{S}}}}}}}}}_{1},{{{{{{{{\rm{S}}}}}}}}}_{2},\ldots {{{{{{{{\rm{P}}}}}}}}}_{i}\ldots {{{{{{{{\rm{S}}}}}}}}}_{n}{{{{{{{{\rm{S}}}}}}}}}_{n+1}\right\rangle$ where the single $\left|{{{{{{{\rm{P}}}}}}}}\right\rangle$-excitation resides on position *i*. This subspace is simulated by choosing *n* particles from the ensemble under blockade condition (representing the seed $\left|{{{{{{{\rm{S}}}}}}}}\right\rangle$-excitations) and an additional, randomly positioned particle (representing the $\left|{{{{{{{\rm{P}}}}}}}}\right\rangle$-excitation). For a set of combinations of the two free parameters, i.e., the number of seed excitations *n* and the blockade radius *r*_*B*_, the eigenvectors $|{\xi }_{j}\rangle$ and eigenvalues *E*_*j*_ of the Hamiltonian Eq. (2) in the considered subspace are numerically determined for 10^5^ random realizations. While the dipole–dipole interaction ${C}_{3}={d}^{2}/(4\pi {\epsilon }_{0})\left(1-3{\cos }^{2}(\theta )\right)$ is calculated from the dipole matrix element *d*, the van der Waals interaction coefficient (${C}_{6}^{{{{{{{{\rm{\downarrow }}}}}}}}}$) is obtained by fitting to pair-state potentials from an exact diagonalization of the many-level system^39^. Obviously, in the single-excitation subspace, the van der Waals interaction between $\left|{{{{{{{\rm{P}}}}}}}}\right\rangle$-states in Eq. (2) vanishes, i.e., ${C}_{6}^{{{{{{{{\rm{\uparrow }}}}}}}}}=0$.

Since the Rabi coupling Ω_P_ is much smaller than the interaction energy of the probed states, we directly couple to the eigenstates of the system. Starting from an initial state $\left|G\right\rangle =\left|SS..Sg\right\rangle$ where the *n* + 1st atom is still in the ground state, the dipole coupling to the eigenstate $|{\xi }_{j}\rangle$ is given by

4 $$\left\langle G| \hat{d}| {\xi }_{j}\right\rangle 	=\; \mathop{\sum }\limits_{i=1}^{n+1}\left\langle G| {\hat{d}}_{n+1}| i\right\rangle \left\langle i| {\xi }_{j}\right\rangle \\ 	 =\; \left\langle G| {\hat{d}}_{n+1}| n+1\right\rangle \left\langle n+1| {\xi }_{j}\right\rangle \\ 	 =\; {d}_{{{{{{{{\rm{gP}}}}}}}}}\left\langle n+1| {\xi }_{j}\right\rangle ,$$

where we inserted unity in the one-excitation subspace and *d*_gP_ is the single-particle dipole matrix element for coupling a 5*S* ground state atom to the Rydberg P-state. Thus, the resulting eigenvalue spectrum of the Hamiltonian is projected onto $\left|n+1\right\rangle$ to obtain the normalized simulated spectra ${\chi }_{n,{r}_{B}}(\nu )={\sum }_{{E}_{j}\approx h\nu }| \langle n+1| {\xi }_{j}\rangle {| }^{2}$ for fixed parameters *n* and *r*_*B*_. Finally, the statistical nature of the seed excitation process provides a Poisson distributed number of seeds *n* across multiple realizations $p(n)={\bar{n}}^{n}{e}^{-\bar{n}}/n!$, with the average seed excitation number $\bar{n}$. This is taken into account in the simulation by taking the Poisson weighted sum of the calculated spectra ${\bar{\chi }}_{\bar{n},{r}_{B}}(\nu )=\mathop{\sum }\nolimits_{i = 0}^{\infty }p(i){\chi }_{i,{r}_{B}}(\nu )$. The summation is truncated at *i* = 18 in our simulations. The simulated spectra ${\bar{\chi }}_{\bar{n},{r}_{B}}(\nu )$ are fitted to the measured spectra by varying the average number of seed excitations $\bar{n}$, the blockade radius *r*_*B*_ and the amplitude *A*, using a least-squares method. The *p*(0) contribution of the Poisson distribution takes an exceptional role here because in absence of seed excitations Rydberg molecules have a strong influence on the spectral shape. Thus, ${\chi }_{0,{r}_{B}}(\nu )$ is modeled with the experimentally obtained non-interacting spectrum instead of a Lorentzian line shape.

### Localization–delocalization crossover

To identify indications of a localization–delocalization crossover based on eigenstate properties we first calculate the coherence Eq. (3) for each eigenvector ${|{\xi }_{j}\rangle }_{l}=\mathop{\sum }\nolimits_{i = 1}^{{n}_{l}+1}{c}_{i}^{j}{\left|i\right\rangle }_{l}$ obtained according to the previous section. The index *l* = {*n*, *r*_B_} denotes combinations of the two free parameters, a number of seed excitations *n* and blockade radius *r*_*B*_. For each parameter set, the eigenstates are binned by eigenenergy and for each bin the conditional probability *P*_*L*_(Δ_P_, *n*) to find a localized dimer state under the condition that excitation at this particular energy *E* occurs is calculated. Note that this is different from the probability to find a localized eigenstate since the probability to couple to the state via the (*n* + 1)st atom is taken into account. Localized states are defined as states with a coherence *C* < *C*_thresh_ = 2. As this condition is fulfilled trivially for systems with maximally 3 particles, we extend the notion of localization to the spatial extent of the states

5 $$S({|{\xi }_{j}\rangle }_{l})=2{\left(\mathop{\sum }\limits_{i = 1}^{{n}_{l}+1}| {c}_{i}^{j}{| }^{2}| {{{{{{{{\bf{x}}}}}}}}}_{i}-{{{{{{{{\boldsymbol{\mu }}}}}}}}}_{{\xi }_{j}}{| }^{2}\right)}^{1/2}$$

around the centroid ${{{{{{{{\boldsymbol{\mu }}}}}}}}}_{{\xi }_{j}}=\mathop{\sum }\nolimits_{i = 1}^{{n}_{l}+1}| {c}_{i}^{j}{| }^{2}{{{{{{{{\bf{x}}}}}}}}}_{i}$, with the random spatial site distribution **x**_*i*_. We define a localized pair state to be confined both in Hilbert space (*C* < 2) and in real space (*S* < *r*_*B*_).

Expressing the localization condition with Heaviside functions $L(|{\xi }_{j}\rangle )={{\Theta }}(2-C(|{\xi }_{j}\rangle ))\,{{\Theta }}\,({r}_{B}-S(|{\xi }_{j}\rangle ))$ the conditional probability *P*_*L*_(Δ_P_, *n*) is equivalent to the expected value of $L(|{\xi }_{j}\rangle )$ within the corresponding energy bin

6 $${P}_{L}({{{\Delta }}}_{{{{{{{{\rm{P}}}}}}}}},n) =P(|{\xi }_{j}\rangle {{{{{{{\rm{localized}}}}}}}}\ | \ |{\xi }_{j}\rangle {{{{{{{\rm{excited}}}}}}}}\cap {E}_{j}\approx h{{{\Delta }}}_{{{{{{{{\rm{P}}}}}}}}})\\ ={\langle L(|{\xi }_{j}\rangle )\rangle }_{{E}_{j}\approx h{{{\Delta }}}_{{{{{{{{\rm{P}}}}}}}}}}=\mathop{\sum}\limits_{{E}_{j}\approx h{{{\Delta }}}_{{{{{{{{\rm{P}}}}}}}}}}L(|{\xi }_{j}\rangle )\frac{| {c}_{n+1}^{j}{| }^{2}}{{\chi }_{n,{r}_{B}}({{{\Delta }}}_{{{{{{{{\rm{P}}}}}}}}})}.$$

Indications for a localization–delocalization crossover based on the eigenenergies are extracted from the scaling of the spectral density. We estimate the energies Δ_CO_ where the crossover of the spectral density ${\chi }_{n,{r}_{B}}({{{\Delta }}}_{{{{{{{{\rm{P}}}}}}}}})$ to ∣Δ_P_∣^−2^ scaling occurs by assuming an algebraic scaling ${\chi }_{n,{r}_{B}}\propto {{{\Delta }}}_{{{{{{{{\rm{P}}}}}}}}}^{-\alpha }$ with a slowly varying energy dependent exponent *α*(Δ_P_). The scaling exponent is extracted by numerical differentiation

7 $$\frac{\partial {\tilde{\chi }}_{n,{r}_{B}}}{\partial {{{\Delta }}}_{{{{{{{{\rm{P}}}}}}}}}}\frac{{{{\Delta }}}_{{{{{{{{\rm{P}}}}}}}}}}{{\tilde{\chi }}_{n,{r}_{B}}}\approx -\alpha ({{{\Delta }}}_{{{{{{{{\rm{P}}}}}}}}}),$$

where ${\tilde{\chi }}_{n,{r}_{B}}$ denotes an approximated spline of degree 5 that is used instead of the data to ensure numerical stability. To facilitate the convergence of the polynomial approximation, we shift the negative scaling exponents by multiplying the spectral density with ${{{\Delta }}}_{{{{{{{{\rm{P}}}}}}}}}^{2}$ and fit the spline to $\epsilon ({{{\Delta }}}_{{{{{{{{\rm{P}}}}}}}}})={\chi }_{n,{r}_{B}}{{{\Delta }}}_{{{{{{{{\rm{P}}}}}}}}}^{2}$. We checked that for all simulated spectra *ϵ*(∣Δ_P_∣) is monotonically increasing and asymptotically approaches a constant value. Thus, the spectral decay is slower than ∝∣Δ_P_∣^−2^ for all Δ_P_, warranting the above assumptions. The crossover energies Δ_CO_ are estimated by a threshold scaling exponent of *α*(Δ_CO_) = 1.7.

## Supplementary information

Supplementary Information

## Data Availability

The raw data from our experiments is available under 10.26204/data/4.

## Code availability

The code that supports the numerical findings of this study is available from the corresponding author upon request.
