# Supplementary material for: Experimental realization of a 3D random hopping model
Source: Nat Commun. 2021 Nov 30;12:6976. doi: 10.1038/s41467-021-27243-2 (PMC8632899; doi:10.1038/s41467-021-27243-2)
Supplement: Supplementary file 1 — Supplementary Information [file 41467_2021_27243_MOESM1_ESM.pdf]

Supplementary Information for Experimental realization of a 3D random hopping model

Carsten Lippe[1], Tanita Klas[1], Jana Bender[1], Patrick Mischke[1], Thomas Niederprüm[1], and Herwig Ott[1,*]

[1]Department of Physics and Research Center OPTIMAS, Technische Universität Kaiserslautern, 67663 Kaiserslautern, Germany

[*]Corresponding author: ott@physik.uni-kl.de

November 4, 2021

Supplementary Note 1: C_6 to on-site energy mapping

The C_6 terms

$$\sum_{\nu=\downarrow,\uparrow}\sum_{i<j}^{n}\frac{C_6^{\nu}}{R_{ij}^6}\hat{n}_i^{\nu}\hat{n}_j^{\nu} \tag{1}$$

in Eq. (2) can be mapped onto an on-site energy term $\sum_{i=1}^{n}\varepsilon_i\hat{\sigma}_i^z$ of the spin model if the n-body Hilbert space is restricted to the subspace that is spanned by the states $|i\rangle = |\downarrow_1,\downarrow_2,\ldots,\uparrow_i,\ldots,\downarrow_n\rangle$ that carry only a single $|\uparrow\rangle$-excitation at atom i.

In this basis, the action of the number operators $\hat{n}_i^{\nu}$ can be expressed with the Kronecker δ symbols as $\hat{n}_i^{\downarrow}|j\rangle = (1-\delta_{ij})|j\rangle$ and $\hat{n}_i^{\uparrow}|j\rangle = \delta_{ij}|j\rangle$. Since $\hat{n}_i^{\uparrow}\hat{n}_j^{\uparrow}|k\rangle = \delta_{ik}\delta_{jk}|j\rangle \equiv 0$ for $i \neq j$, we see that, as expected, the contribution of the $C_6^{\uparrow}$-term which describes the interaction between multiple $|\uparrow\rangle$-excitations vanishes in the single-$|\uparrow\rangle$ subspace.

Now, we consider $|\chi\rangle = \sum_{i=1}^{n} c_i|i\rangle$ with $\sum_{i=1}^{n}|c_i|^2 = 1$ to be an arbitrary normalized state from the single excitation subspace. The action of the $C_6^{\downarrow}$-term from Supplementary Eq. (1) on $|\chi\rangle$ then yields

$$\begin{aligned}\sum_{i<j}^{n}\frac{C_6^{\downarrow}}{R_{ij}^6}\hat{n}_i^{\downarrow}\hat{n}_j^{\downarrow}|\chi\rangle &= \frac{1}{2}\sum_{i\neq j}^{n}\frac{C_6^{\downarrow}}{R_{ij}^6}\sum_{k=1}^{n}c_k(1-\delta_{ik})(1-\delta_{jk})|k\rangle \\ &= \frac{1}{2}\sum_{i\neq j}^{n}\frac{C_6^{\downarrow}}{R_{ij}^6}\sum_{k=1}^{n}c_k(1-2\delta_{ik})|k\rangle\,, \end{aligned} \tag{2}$$

because the product term $\sum_{i\neq j}^{n}\delta_{ik}\delta_{jk}$ vanishes.

Defining position dependent energies $\varepsilon_i = -\frac{1}{2}\sum_{j\neq i}^{n}\frac{C_6^{\downarrow}}{R_{ij}^6}$ and using the identiy $\hat{\sigma}_i^z|k\rangle = (2\delta_{ik}-1)|k\rangle$ we can reformulate Supplementary Eq. (2) in terms of Pauli matrices

$$\begin{aligned}\sum_{i<j}^{n}\frac{C_6^{\downarrow}}{R_{ij}^6}\hat{n}_i^{\downarrow}\hat{n}_j^{\downarrow}|\chi\rangle &= -\frac{1}{2}\sum_{i=1}^{n}\sum_{j\neq i}^{n}\frac{C_6^{\downarrow}}{R_{ij}^6}\hat{\sigma}_i^z\sum_{k=1}^{n}c_k|k\rangle \\ &= \sum_{i=1}^{n}\varepsilon_i\hat{\sigma}_i^z|\chi\rangle\,. \end{aligned}$$

In the single excitation subspace, the model Hamiltonian Eq. (2) therefore takes the form of an XY model in a longitudinal random field Eq. (1).

Supplementary Note 2: Coherence of the system

When the interaction energy $V_{ij}^{\mathrm{dd}} \gg \frac{\hbar}{\tau_{\mathrm{d}}}$ dominates the inverse dephasing time τ_{d} and the probing remains weak, i.e. $\Omega_{\mathrm{P}} \ll V_{ij}^{\mathrm{dd}}$, we directly excite the coherent many-body state formed by the n seeds and one additional atom excited by the probe pulse. The off-diagonal nature of the dipole–dipole interaction intrinsically provides coherence on the two particle level. In order to destroy this coherence any decoherence process (laser noise, atomic motion, internal decay) must be of similar magnitude.

A lower bound for the decoherence time $\tau_{\mathrm{d}} = 150\,\mathrm{ns}$ is taken from the pulse length limited linewidth $\Delta\nu \approx 1\,\mathrm{MHz}$ in the measured reference spectrum. Note that for longer exposure times, we have previously observed a linewidth of less than 300 kHz. For the blockade radius $r_B \approx 3\,\mu\mathrm{m}$, we find a hopping time $\hbar/J = 30\,\mathrm{ns}$. This allows for at least 5 hopping events in each spatial direction, thus essentially covering the whole atomic cloud.

Supplementary Note 3: Signal evaluation

Spectra. We determine the average number of excitations created by the pump and the probe laser relying on the method depicted in Fig. 2a–b. By making a reference measurement (Fig. 2a), where the probe pulse is delayed by $\tau = 300\,\mu\mathrm{s}$, we obtain the signal created by the seeds alone (gray area) and the spectroscopic signal of the $|\uparrow\rangle$-excitations without the presence of the seeds (green area). For the much smaller pump–probe delay $\tau = 1\,\mu\mathrm{s}$, which we use for the interacting spectra, the signals generated

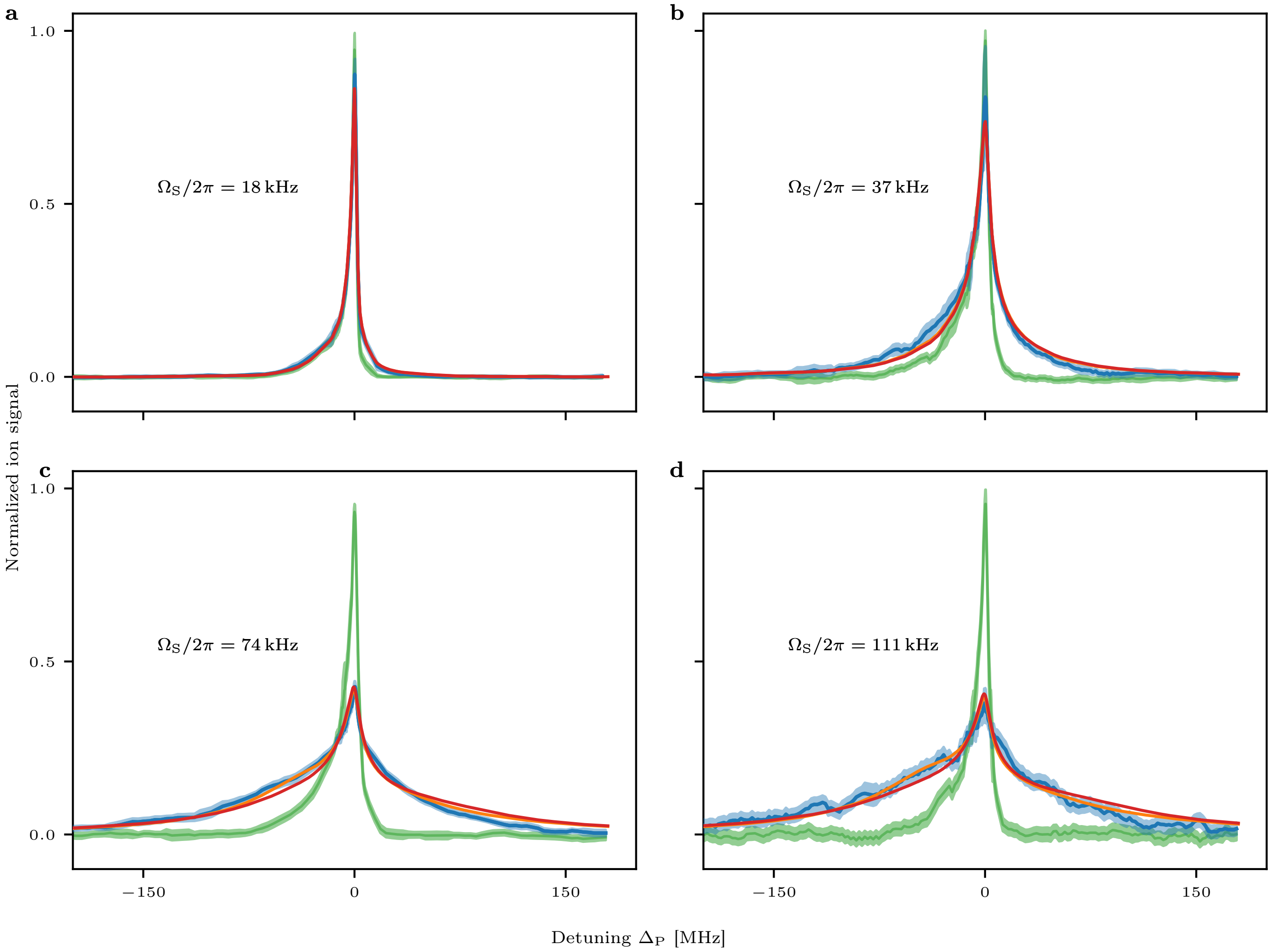

Supplementary Figure 1: Influence of the on-site energy. Spectra for the excitation of the $|51P_{3/2}\rangle$-state after the creation of different numbers of initial seeds in the $|51S_{1/2}\rangle$-state with two-photon Rabi frequencies Ω_S **a** $2\pi \times 18\,\mathrm{kHz}$, **b** $2\pi \times 37\,\mathrm{kHz}$, **c** $2\pi \times 74\,\mathrm{kHz}$, **d** $2\pi \times 111\,\mathrm{kHz}$. The shaded areas denote the standard error of the mean. The same spectra are already shown in Fig. 2**c**–**f**. In addition to the fitted numerical model with ($C_6 \neq 0$, orange), a comparison for a model without ($C_6 = 0$, red) on-site energy is shown. The non-interacting spectra (green) are given as reference.

by both excitation pulses overlap (Fig. 2b). We therefore use the knowledge of the isolated seed contribution from the reference measurement (gray area) and, by subtraction, isolate the contribution from the $|\uparrow\rangle$ excitation (blue area).

The integrated number of events in the blue area comprises our measurement signal for the interacting case ($|\uparrow\rangle$-excitation in the presence of the seeds) and the integrated number of events in the green area is the measurement signal for the reference spectra ($|\uparrow\rangle$-excitation in the absence of seeds).

Lifetimes. The time-resolved signal stemming from probe excitations $I(t)$ is obtained with a similar evaluation scheme as described in the previous section. For each parameter combination (Ω_S, Δ_P) we subtract the time-resolved non-interacting signal from the interacting measurement. The ionization processes present in our setup can create atomic Rb^+ or molecular Rb_2^+ ions, which we can distinguish by their arrival time on the detector. Thus, by choosing a proper window of arrival times (70 µs up to $\sqrt{2} \times 70\,\mu\mathrm{s} \approx 100\,\mu\mathrm{s}$ after the excitation pulses) we can isolate the time-resolved signal of the Rb^+ ions. Near resonance $|\Delta_P| \approx 0\,\mathrm{MHz}$ we verify that the decay of this signal is exponential. For increasing detuning, however, the rapidly decreasing signal strength makes fitting an exponential function challenging. Thus, to obtain lifetimes in a numerically more stable way, we make use of the relation

$$\int_0^\infty A \exp(-t/\tau_{\mathrm{Rb}^+})dt = A\tau_{\mathrm{Rb}^+} \qquad (3)$$

between the integral signal and its amplitude A and lifetime τ_{Rb^+}. The lifetime is now obtained by dividing the integrated Rb^+ signal $\int_{70\,\mu\mathrm{s}}^{100\,\mu\mathrm{s}} I(t')dt' \approx \int_0^\infty A\exp(-t/\tau_{\mathrm{Rb}^+})dt$ by its amplitude A.

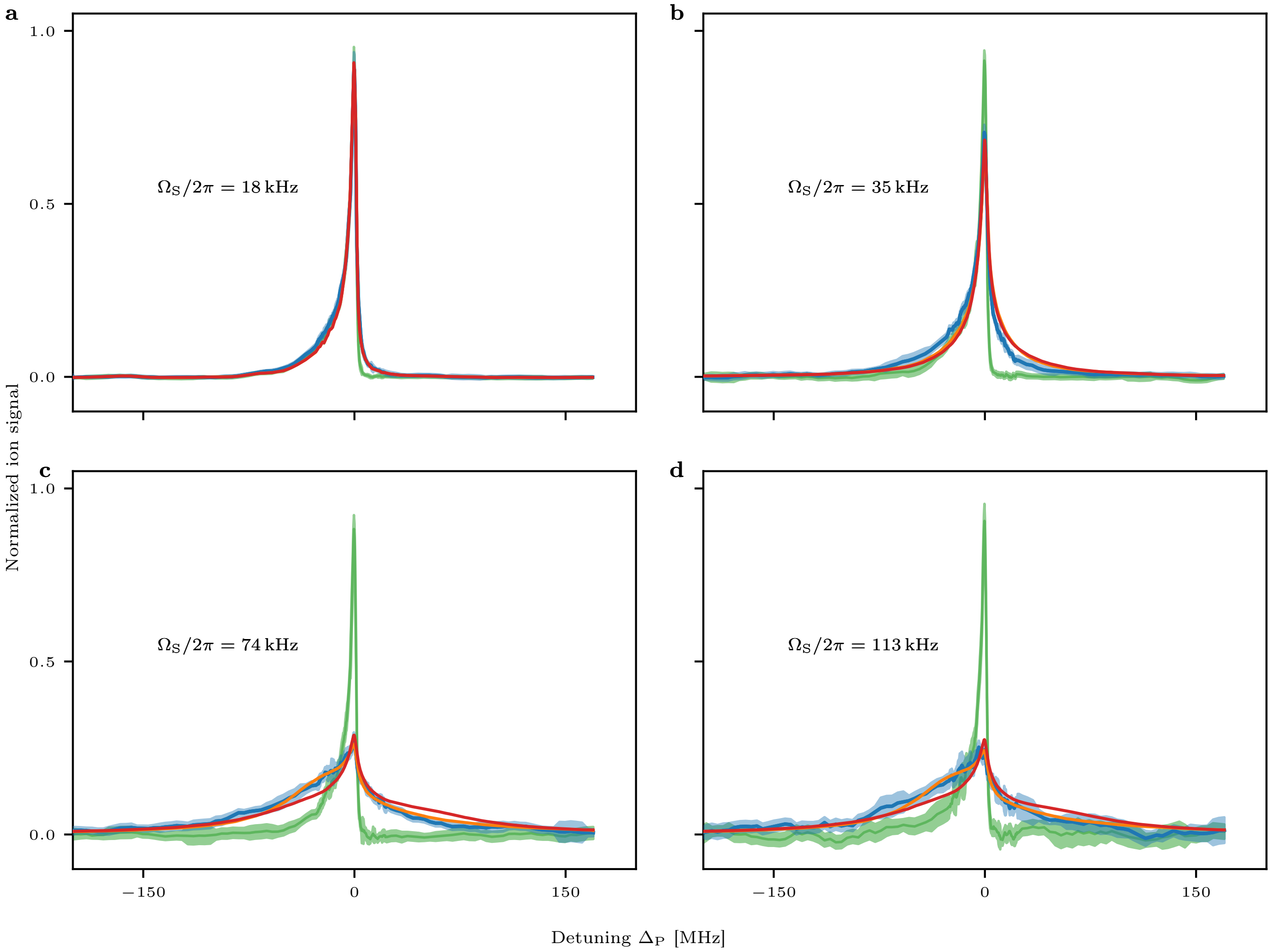

Supplementary Figure 2: Influence of the Rydberg state. Spectra for the excitation of the $|51P_{1/2}\rangle$-state after the creation of different numbers of initial seeds in the $|51S_{1/2}\rangle$-state (blue) are fitted with the numerical model with ($C_6 \neq 0$, orange) and without ($C_6 = 0$, red) on-site energy term. The non-interacting spectra (green) are given as reference. We extract average coherently coupled seed excitation numbers $\bar{n}$ of **a** 0.3, **b** 3.2, **c** 8.4 and **d** 8.9.

Supplementary Note 4: Influence of the on-site energy

The van der Waals interactions between the $|\downarrow\rangle$ seeds appearing in Eq. (2) can be mapped to the on-site energy term of Eq. (1). Due to the positional disorder of the seed excitations, these on-site energy terms have a randomized magnitude – although not completely random due to fundamental geometric constraints and the blockade condition: The distance between any two seeds has a lower bound given by the Rydberg blockade. Only the position of the atom that is additionally excited by the probe pulse is not limited by Rydberg blockade, and may therefore have a smaller distance to other involved atoms. Additionally the triangular inequality and it's generalizations impose limits (which appear as correlations) on the randomness of the individual distances. For instance, given three atoms A, B and C, the distance $\overline{AC}$ between atoms A and C is limited by the distances $\overline{AB}$ and $\overline{BC}$. The system simultaneously realizes disorder of the hopping strength as well as of the on-site energy. However, the dominant energy scale is set by the dipole–dipole interaction and thus the effect of the on-site energy disorder is suppressed. In order to quantify the effect of the on-site energy term in our measured spectra, we compare the experimental results to our numerical model with ($C_6 \neq 0$) and without ($C_6 = 0$) the on-site energy term. The comparisons shown in Supplementary Fig. 1 and, for a measurement with a different $|\uparrow\rangle$-state, in Supplementary Fig. 2 reveal that the model including the on-site energy term (orange) achieves a better agreement with the data than the model without (red). While our resolution is sufficient to see the effect of the on-site energy, we also see that its contribution on the overall broadening and the line shape is small.

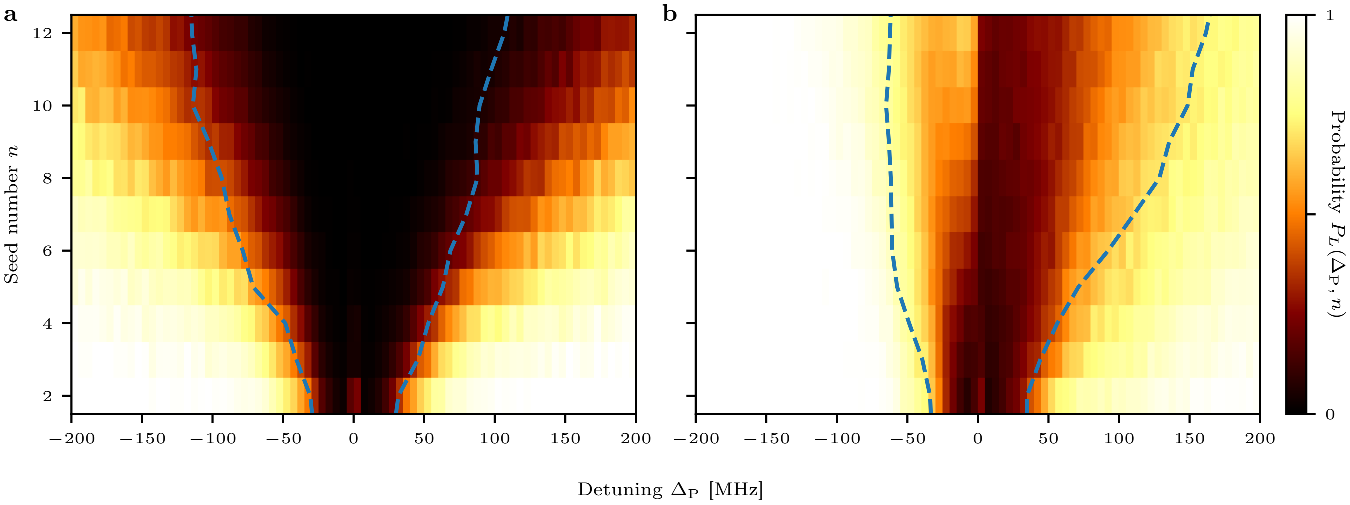

Supplementary Figure 3: Localization–delocalization crossover. We plot the probability to find a localized state $p_{E,n}$ **a** for a pure random XY model and **b** with additional C_6 interaction between the seeds as realized in the experiment. In contrast to Fig. 3 in the main text, here the probe atom is excited into another fine structure state, the $|51\mathrm{P}_{1/2}\rangle$-state. The regime of predominantly delocalized states spreads for increasing number of seeds n, shifting the localization–delocalization crossover to larger energies. The estimated energies where the corresponding spectra show a transition towards algebraic $|\Delta_\mathrm{P}|^{-2}$ scaling are denoted by dashed lines.

Supplementary Note 5: Influence of the Rydberg state

Spectrum. To study the effect of the relative strength of the hopping and the on-site energy term we repeated the same experiment with a different Rydberg state. We keep the on-site energy identical by choosing the same seed state $|\downarrow\rangle = |51\mathrm{S}_{1/2}\rangle$. The probing is realized with the other 51P fine structure state $|\uparrow'\rangle = |51\mathrm{P}_{1/2}\rangle$. In this state the C_3 coefficient is approximately half the size of the C_3 coefficient of $|51\mathrm{P}_{3/2}\rangle$ investigated in the main text. The simulations with on-site energy ($C_6 \neq 0$) shown in Supplementary Fig. 2 (orange) display the same level of agreement to the measured data (blue) as those for $|51\mathrm{P}_{3/2}\rangle$. For this set of measurements we obtain a slightly smaller blockade radius $r_B = 3\,\mu\mathrm{m}$. Due to the weaker hopping strength the influence of the on-site energy term on the lineshape is increased. As expected, this leads to larger relative deviations of the model with absent on-site energy ($C_6 = 0$, red).

Localization. To also study the effect of the relative strength of the hopping and the on-site energy term on the localization properties, we repeated the simulations for another fine structure state $|\uparrow'\rangle = |51\mathrm{P}_{1/2}\rangle$. The seed state was kept constant $|\downarrow\rangle = |51\mathrm{S}_{1/2}\rangle$. As stated above, for this state combination the C_3 coefficient is approximately halved. The phase diagram for the pure random XY model (Supplementary Fig. 3a) exhibits a substantially broader delocalized regime than the one with additional C_6 interaction (Supplementary Fig. 3b). Since in the weak probing limit the C_6 interaction can be mapped to a random on-site energy, the spectral narrowing can be interpreted as a manifestation of Anderson localization. It should also be noted, that the same level of agreement between the transition energies and the phase diagram is reached as for the other fine structure state.

The comparison between Supplementary Fig. 4a and Supplementary Fig. 4b shows the different spectral narrowing for the two studied finestructure states, i.e. for different relative strength of the the on-site and the hopping disorder. Each of the diagrams shows equiprobability lines $L_{0.5} = \{(E,n)|p_{E,n} = 0.5\}$ with (orange) and without (blue) C_6 interaction as can be extracted from Fig. 3 and Supplementary Fig. 3. Since, due to the smaller C_3 coefficient, the relative on-site disorder strength is twice as large as for the $|\uparrow'\rangle = |51\mathrm{P}_{1/2}\rangle$ state as compared to the $|\uparrow\rangle = |51\mathrm{P}_{3/2}\rangle$ state, we consequently see a substantially stronger spectral narrowing for the former.

Supplementary Note 6: Collective reduction of the blockade radius

The Rydberg blockade radius r_B for a pair of atoms is given as the distance below which the van der Waals interaction C_6/r_B^6 between the atoms exceeds the Rydberg excitation linewidth $w = \sqrt{4\Omega^2\gamma/\Gamma_s + \gamma^2}$ [1, 2],

$$r_B = \sqrt[6]{\frac{C_6}{\hbar w}}. \tag{4}$$

The linewidth depends on the driving strength Ω and the total relaxation rate $\gamma = \frac{1}{2}(\Gamma_s + \Gamma_d)$ is composed of spontaneous decay with a rate $\Gamma_s = 2\pi \times 7\,\mathrm{kHz}$ and dephasing Γ_d which includes the excitation laser linewidths, motional dephasing and residual fluctuations of the electric field. We estimate the total dephasing rate to be $\Gamma_d = 2\pi \times 500\,\mathrm{kHz}$.

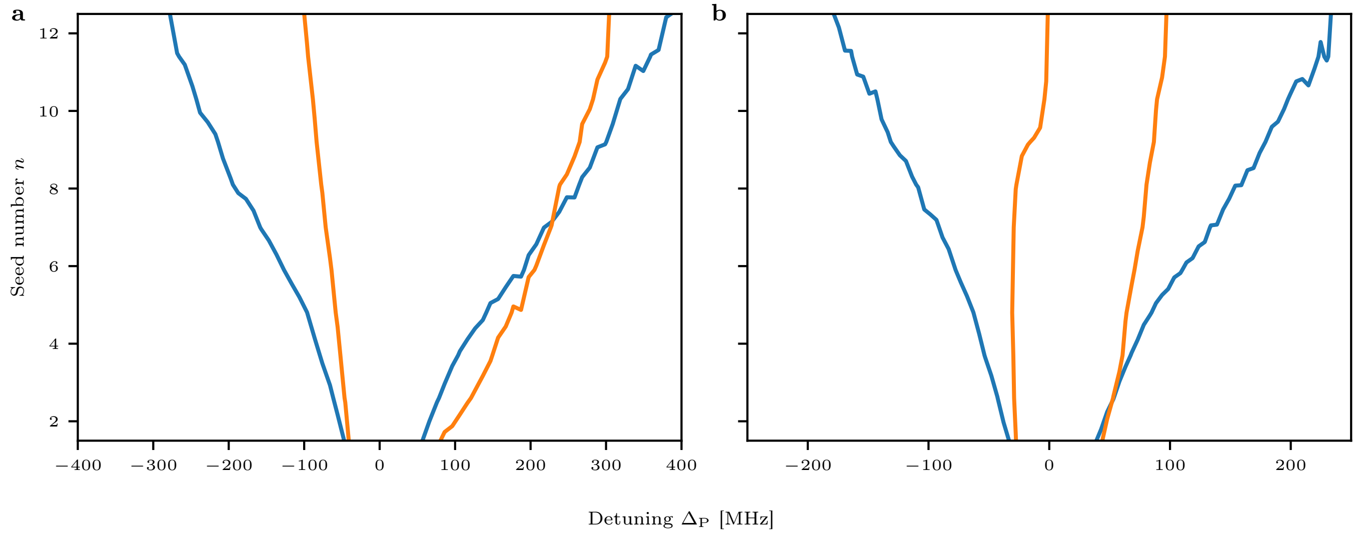

Supplementary Figure 4: Influence of the Rydberg state on localization. Comparison of the equiprobability lines $L_{0.5}$ for the pure random XY model (blue) and with additional C_6 interaction (orange) for a probe atom in **a** the $|51\mathrm{P}_{3/2}\rangle$-state and **b** the $|51\mathrm{P}_{1/2}\rangle$-state. The energy range where the states are predominantly delocalized is substantially narrower in the model with C_6 interaction than in the pure random XY model. Additionally, the relative difference between both models is more pronounced for the $|51\mathrm{P}_{1/2}\rangle$-state. Note that the energy scales are different.

In the dense atomic samples investigated here the Rydberg seed excitation is shared coherently among a large number N of atoms within the Rydberg blockade sphere forming an effective two-level system with collectively enhanced Rabi frequency $\sqrt{N}\Omega$. These so-called superatoms [3] require us to consider additional effects to estimate the blockade radius correctly. In the realized strong driving, strong dephasing regime ($\Gamma_s \ll \Gamma_d, \Omega$) the collective Rabi frequency broadens the superatom excitation linewidth to $w_{SA} = \sqrt{2N\Omega^2\gamma/\Gamma_s + \gamma^2} \approx \sqrt{2N\Omega^2\gamma/\Gamma_s}$, which depends on the number of atoms N inside the superatom [4]. $N = \frac{4}{3}\pi r_B^3 \rho$ depends on the blockade volume and the atomic density ρ. This effect leads to a reduction of the blockade radius. Substituting the superatom linewidth into Supplementary Eq. (4) yields

$$r_B = \left(\frac{C_6}{\hbar\sqrt{2\Omega^2 \frac{\gamma}{\Gamma_s} \frac{4}{3}\pi r_B^3 \rho}} \right)^{\frac{1}{6}}. \tag{5}$$

Solving for r_B finally gives an expression for the blockade radius in a three-dimensional system [5]

$$r_B = \left(\frac{C_6}{\hbar\sqrt{\frac{4}{3}\pi\rho}\sqrt{2\Omega^2\gamma/\Gamma_s}} \right)^{\frac{2}{15}}. \tag{6}$$

Thus, for the experimentally applied range of coupling strengths Ω we estimate blockade radii between 2.6 µm and 3.3 µm under the assumption of homogeneously distributed atoms inside the trapping volume.

Supplementary Note 7: Notion of localization

In the main text, we introduce the coherence C as a measure for the localization of the system. However, the single criterion $C < 2$ to identify localized states does not work for very small system sizes since they fulfill the criterion trivially. Thus, we have to extend our localization criterion to consistently characterize the localization for all system sizes. Thus, we additionally require a localized state to have a spatial extent $S < r_B$ smaller than the blockade radius.

It is important to note that criterion on the coherence $C < 2$ strictly only requires a localization in Hilbert space that is not necessarily connected to a real space localization. This becomes particularly evident in multilevel systems where an eigenstate could delocalize in Hilbert space over many states within one particle while staying spatially localized. But even in our case of the two-level system this discrepancy arises because the localized dimer state is in fact delocalized itself. The most localized state with $C = 1$ (localized dimer) is spread over exactly two bare states, which in our effective spin model corresponds to two sites in real space. These sites, however, could in principle be arbitrarily far apart. This contradicts a naive picture of localization that would also expect a confinement in real space. In Supplementary Fig. 5 we investigate different notions of localization. To this end, we compare the probability to find a state with small spatial extent (Supplementary Fig. 5a) to the probability of a state with small coherence (Supplementary Fig. 5b). Supplementary Fig. 5c combines both conditions. Indeed, we see that the probability map is identical to Supplementary Fig. 5b for $n \gtrsim 3$. We thus conclude, that all states that don't trivially fulfill the Hilbert

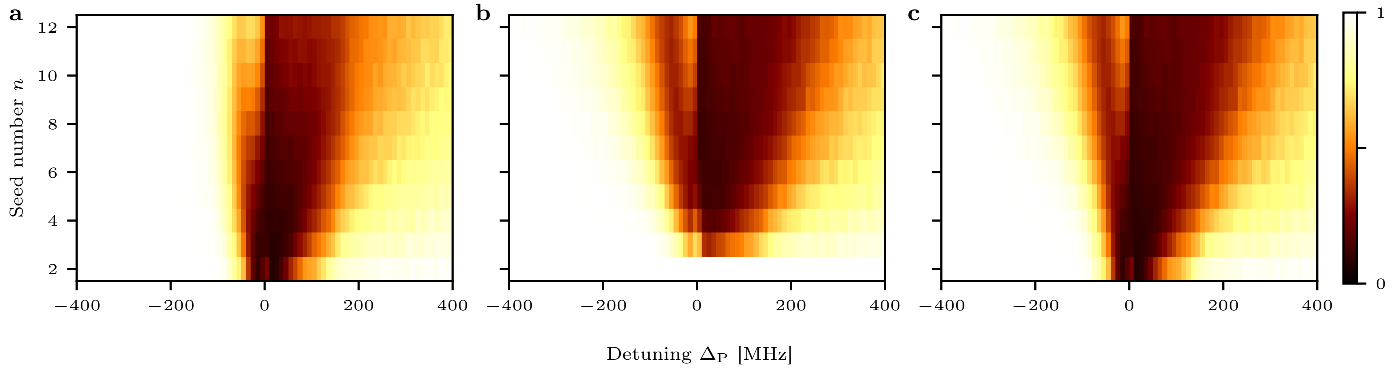

Supplementary Figure 5: Different notions of localization. We plot the probability to find a state that **a** fulfills the real space localization condition $S < r_B$, **b** fulfills the Hilbert space localization condition $C < 2$ and **c** fulfills both conditions. The probability maps **b** and **c** are almost identical, indicating that real space localization is required for Hilbert space localization.

space localization condition, are already localized in real space.

Supplementary Note 8: Localization probabilities and $|\Delta_\mathrm{P}|^{-2}$ scaling

In Supplementary Fig. 6 we show how the localization-delocalization from Fig. 3 connects to the spectra as shown in Fig. 4. The mathematical calculation for obtaining the detuning Δ_CO is presented in the Methods section.

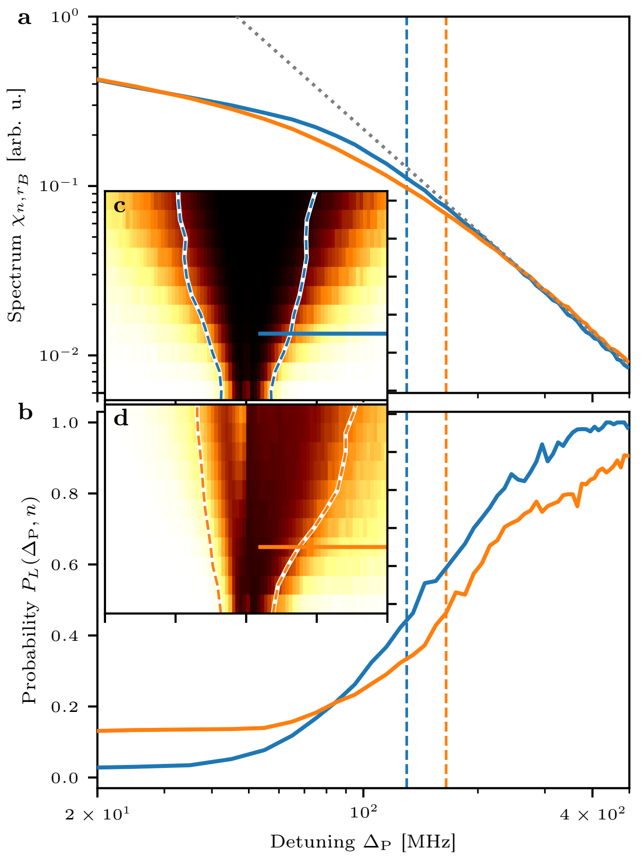

Supplementary Figure 6: Comparison between spectra χ and probability of localization P_L. **a** shows the simulated spectra for $n = 4$ seeds with (blue) or without (orange) C_6 interaction. The dashed lines indicate the detuning Δ_{CO} where the transition towards $|\Delta_P|^{-2}$ scaling (dotted gray line) sets in. For the calculation of these detunings see Methods. **b** shows the probability P_L to find a localized state. The detuning Δ_{CO} calculated from the spectra matches the crossover to localized states. The insets **c** and **d** show the Probability P_L for different seed numbers and are identical to Fig. 3, with the position of the cuts shown in **b** marked. The dashed lines show Δ_{CO} from the simulated spectra as shown in **a**.

Supplementary References

[1] Urban, E. *et al.* Observation of Rydberg blockade between two atoms. *Nat Phys* **5**, 110–114 (2009).

[2] Petrosyan, D., Höning, M. & Fleischhauer, M. Spatial correlations of Rydberg excitations in optically driven atomic ensembles. *Physical Review A* **87**, 053414 (2013).

[3] Weber, T. M. *et al.* Mesoscopic Rydberg-blockaded ensembles in the superatom regime and beyond. *Nat Phys* **11**, 157–161 (2015).

[4] Letscher, F., Petrosyan, D. & Fleischhauer, M. Many-body dynamics of holes in a driven, dissipative spin chain of Rydberg superatoms. *New Journal of Physics* **19**, 113014 (2017).

[5] Heidemann, R. *et al.* Rydberg excitation of bose-einstein condensates. *Physical Review Letters* **100**, 033601 (2008).
